# Nucleotide Excision Repair Is Associated with the Replisome and Its Efficiency Depends on a Direct Interaction between XPA and PCNA

**DOI:** 10.1371/journal.pone.0049199

**Published:** 2012-11-13

**Authors:** Karin M. Gilljam, Rebekka Müller, Nina B. Liabakk, Marit Otterlei

**Affiliations:** Department of Cancer Research and Molecular Medicine, Faculty of Medicine, Norwegian University of Science and Technology, Trondheim, Norway; Universita' di Milano, Italy

## Abstract

Proliferating cell nuclear antigen (PCNA) is an essential protein for DNA replication, DNA repair, cell cycle regulation, chromatin remodeling, and epigenetics. Many proteins interact with PCNA through the PCNA interacting peptide (PIP)-box or the newly identified AlkB homolog 2 PCNA interacting motif (APIM). The xeroderma pigmentosum group A (XPA) protein, with a central but somewhat elusive role in nucleotide excision repair (NER), contains the APIM sequence suggesting an interaction with PCNA. With an in vivo based approach, using modern techniques in live human cells, we show that APIM in XPA is a functional PCNA interacting motif and that efficient NER of UV lesions is dependent on an intact APIM sequence in XPA. We show that XPA^−/−^ cells complemented with XPA containing a mutated APIM sequence have increased UV sensitivity, reduced repair of cyclobutane pyrimidine dimers and (6–4) photoproducts, and are consequently more arrested in S phase as compared to XPA^−/−^ cells complemented with wild type XPA. Notably, XPA colocalizes with PCNA in replication foci and is loaded on newly synthesized DNA in undamaged cells. In addition, the TFIIH subunit XPD, as well as XPF are loaded on DNA together with XPA, and XPC and XPG colocalize with PCNA in replication foci. Altogether, our results suggest a presence of the NER complex in the vicinity of the replisome and a novel role of NER in post-replicative repair.

## Introduction

Proper repair of DNA is vital in order to avoid mutations that may cause cancer and other diseases. Cells have therefore evolved numerous pathways to deal with a variety of DNA damage, many of which are associated with the replication machinery [1]–[3]. Xeroderma pigmentosum group A (XPA) is a protein in the Nucleotide excision repair (NER) pathway responsible for removal of a wide range of lesions leading to distortions of the DNA helix, most frequently caused by UV radiation (UVR) from the sun. UVR-induced DNA damage inhibits DNA transcription and replication, leading to S phase delay and, if the damage is left unrepaired, may induce DNA double strand breaks (DSBs) [4], [5]. However, NER has not been directly coupled to the replicative process.

The historically scientific interest in NER has partly been due to the severe clinical phenotype seen in patients with inherited deficiency in this pathway. The NER pathway involves more than 30 proteins and defects in any of the central NER proteins may result in premature aging, neurodegenerative diseases and/or hypersensitivity to UVR. The skin cancer disease xeroderma pigmentosum (XP) is a result of deficiency in any of the seven XP-genes coding for proteins involved in NER. XP patients exhibit more than a 1,000-fold increase in the incidence of sun-induced skin cancer and an increased incidence of internal cancers, primarily in the lung or gastro-intestinal tract [6], [7]. Moreover, 30% of XP patients suffer from neurological diseases in addition to the increased incidents of cancer [8].

Solar UV-B and UV-C radiation generate pyrimidine crosslinks, both cyclobutane pyrimidine dimers (CPDs) and 6-4 photo products (6-4 PPs). Particularly the 6-4 PPs are rapidly recognized by NER [9], [10]. CPDs, however, are less efficiently recognized by NER, but are easily bypassed by the translesion synthesis (TLS) polymerase POLη [11]. These bypassed CPDs are believed to be repaired by NER prior to next round of replication. DNA damage in the actively transcribed strand is recognized by the stalling of the RNA polymerase in a process called transcription coupled (TC) NER, while damage recognized and repaired independent of transcription is called global genome (GG) NER. After damage recognition, TC-NER and GG-NER have similar mechanisms involving dual incision, removal of a 25–30 nucleotide fragment and re-synthesis of the gap. Of the many proteins involved in NER, XPA is indispensable due to its central role in the core incision complex where it is suggested to be the rate limiting factor [12]. XPA is believed to be important for damage verification and the tethering of the NER components to DNA, although its exact role is still unclear [10]. Nonetheless, it is the only NER protein that is present in all the steps from damage verification to the repair synthesis [13]. Among XPÁs many interaction partners are replication protein A (RPA), XPA-binding protein 1 and 2 (XAB1 and XAB2), transcription factor II H (TFIIH), XPC, excision repair cross-complementation group 1 protein (ERCC1), and the checkpoint kinase ATR [14]–[23]. In addition XPA interacts with both DNA and itself, forming homodimers [24]–[26].

Interestingly, we found XPA to contain the new proliferating cell nuclear antigen (PCNA) interaction motif called AlkB homolog 2 PCNA interacting motif (APIM) (K/R-F/Y/W-[L/I/V/A]×2-K/R) [27], suggesting a previously unidentified direct interaction between XPA and PCNA. PCNA is essential for numerous cellular processes including DNA replication and repair [28]. PCNA is also an essential component of NER where it plays a role in mediating repair synthesis after dual incision [29], [30]. Numerous proteins contain conserved sequences that fit with the PCNA interacting peptide (PIP)-box (QxxL/I/MxxHF/DF/Y) [31] or the APIM consensus sequence [27], [32] (http://tare.medisin.ntnu.no/pcna/index.php). However, experimentally, only few of the APIM sequences in the long list of proteins are proven to be functional. In this study we show for the first time that XPA directly interacts with PCNA via its APIM sequence, an interaction required for optimal NER. We detect the interaction in replication foci and identify XPA, XPF and XPD on nascent DNA at replication forks in untreated, cycling cells. The presence of NER proteins close to replication forks suggests a novel function of NER in post-replicative repair.

## Results and Discussion

### XPA Interacts with PCNA in Replication Foci

**Figure 1:**
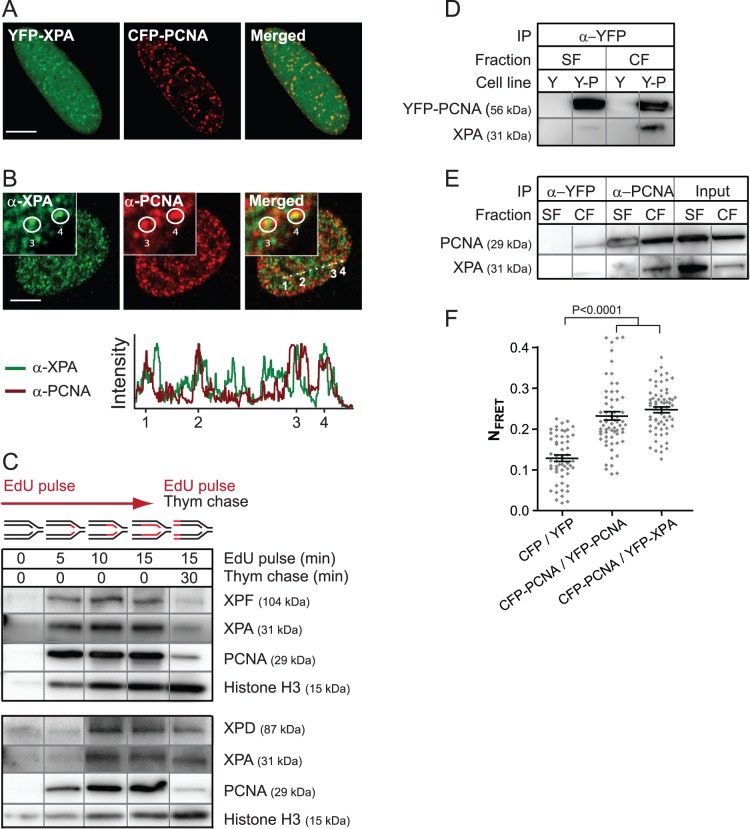
XPA colocalizes and directly interacts with PCNA in replication foci . (A) Overexpressed tagged proteins in live cycling HeLa cells. (B) Immunostained HeLa cells. The intensity of α-XPA and α-PCNA along the line in the merged picture is illustrated in the graph. The inserts show an enlargement of the area close to foci 3 and 4. (A and B) Bar: 5 µm. (C) iPOND from cells labeled with EdU (pulse) before fixation. One sample was additionally followed by a chase in thymidine-containing medium (pulse-chase). The WB shows proteins captured due to EdU proximity. The upper and lower panels are from individual iPOND experiments. All bands within one panel (black frame) are from the same WB, lanes and rows are separated by grey lines (also in D and E). (D) Co-IP of endogenous XPA from HeLa cells stably expressing YFP-PCNA using α-YFP beads. SF: soluble fraction, CF: chromatin-enriched fraction, Y: YFP (negative control), Y-P: YFP-PCNA. (E) Co-IP of endogenous XPA from untransfected HeLa cells using α-PCNA beads (pulling down endogenous PCNA). IP with α-YFP was used as control for unspecific binding to the beads. (F) Normalized FRET (N_FRET_) measurements in HeLa cells. CFP/YFP (vectors only) and CFP-PCNA/YFP-PCNA were used as negative and positive controls, respectively. Detector gain: 800 (YFP), 700 (CFP), 700 (FRET). The P-value is derived by unpaired t-test. Data presented is from three independent experiments (mean ± SEM, n = 55–75).

APIM in XPA (amino acid (aa) 163–167) is found within the suggested DNA binding domain of XPA (aa 138–209) between loop 1 and 2, two regions reported to be highly mobile in solution [27], [33]. We first co-expressed XPA with its potential interaction partner PCNA in untreated HeLa cells and found that YFP-tagged XPA (YFP-XPA) colocalized with CFP-tagged PCNA (CFP-PCNA) in foci resembling replication foci (Figure 1A). Localization of XPA in replication foci was somewhat surprising because an association between the NER pathway and the replication machinery has hitherto not been discussed in the literature. In order to examine whether this observation was an artifact of overexpression, we performed immunofluorescent labeling and iPOND: isolation of proteins on nascent DNA [34], on cells expressing only endogenous proteins. Immunofluorescence indicated, although with lower resolution than in live cell imaging, colocalization between XPA and PCNA in foci likely representing replication foci as judged by the PCNA pattern (Figure 1B). The graph in Figure 1B illustrates the intensity of endogenous XPA and PCNA along the line visualized in the merged picture. Numbers 1 to 4 represent replication foci where a clear colocalization between XPA and PCNA was observed (yellow foci in the inserts). Additionally, many foci showed presence of both XPA and PCNA, but at different fluorescent intensities. No increase in colocalization between XPA and PCNA was seen after UVR (unpublished data). The specificity of the XPA antibody was verified in XPA deficient (XPA^−/−^) fibroblast cells (unpublished data). The new high resolution technique iPOND was employed to further verify the localization of endogenous XPA to the replisome in absence of DNA damage. This method detects proteins in the proximity of newly incorporated 5-ethynyl-2′-deoxyuridine (EdU), hence proteins binding to active replication forks as elegantly showed by Sirbu and colleagues [34], [35]. Cells were treated for 0 to 15 min with EdU (pulse) including one sample where 15 min EdU pulse was followed by thymidine (chase) prior to fixation (Figure 1C). We found that, similar to PCNA (positive control for replication fork proteins), XPA was detected after only 5 min EdU pulse, and more XPA was pulled down in the pulse than in the pulse-chase sample (Figure 1C, upper panel). Similar patterns were observed for XPF and XPD, other proteins in the core incision NER complex. The upper and lower panels show western analysis from individual iPOND experiments where, based on the recruitment pattern of PCNA and XPA, the replication rate at the experiment visualized in the lower panel appears to be lower. Nevertheless, the intensity of XPF and XPD follows the intensity of XPA and PCNA, suggesting that the NER complex, and not only XPA, is associated with the replisome in undamaged freely cycling cells. As a control for the biotin capture, we also stained for Histone H3 known to load to nascent DNA at later time points [35]. We did not detect XPC on the same membrane, neither in input nor capture, suggesting low sensitivity of the antibody used in this study. However, we did detect clear colocalization of both YFP-XPC and YFP-XPG with CFP-PCNA in foci resembling replication foci (Figure S1). Together, these results strongly suggest a presence of NER proteins in the replisome in undamaged cells. This presence would enable NER to execute rapid post-replicative repair following bypass of DNA lesions. ZRANB3, a translocase important for restart of arrested replication forks containing a functional APIM sequence, was also recently found to be in replication foci in absence of DNA damage [32].

Next we examined whether, and from which cell fraction, endogenous XPA could be pulled down with PCNA. Similarly to what was found for human AlkB homolog 2 (hABH2) [27], more XPA could be pulled down by co-immunoprecipitation (IP) from the chromatin-enriched fraction (CF) than from the soluble fraction (SF) (Figure 1D and E), although both PCNA and XPA were abundant in SF (Input, Figure 1E). We obtained the same results in HeLa cells overexpressing PCNA (Figure 1D) as in cells only expressing endogenous PCNA (Figure 1E), and no increase in the amounts of XPA pulled down after UVR was detected (unpublished data). Proteins associated with the replication machinery are likely to be in the CF; hence the co-IP data fits well with the colocalization and iPOND data. The HeLa cell extracts used in these experiments were excessively treated with DNAses and RNAses to abolish any potential binding through DNA.

To examine whether XPA directly interacts, and not only colocalizes, with PCNA, we measured the fluorescence resonance energy transfer (FRET) between the proteins. FRET can only occur when the fluorescent tags are less than 10 nm apart [36], thus the tagged proteins are in close proximity suggesting a direct interaction. The FRET level between YFP-XPA and CFP-PCNA was similar to that detected between YFP-PCNA and CFP-PCNA suggesting that XPA and PCNA are as close as two PCNA monomers within a PCNA trimer (Figure 1F).

In summary, XPA and PCNA interact and at least a sub-fraction of XPA is localized close to active replication forks. Notably, this interaction takes place in untreated cells and there are no detectable differences in colocalization or co-IP after UVR, suggesting that the NER proteins are normal constituents of the replisome.

### The APIM Sequence in XPA is Sufficient and Necessary for a Direct PCNA Interaction

**Figure 2:**
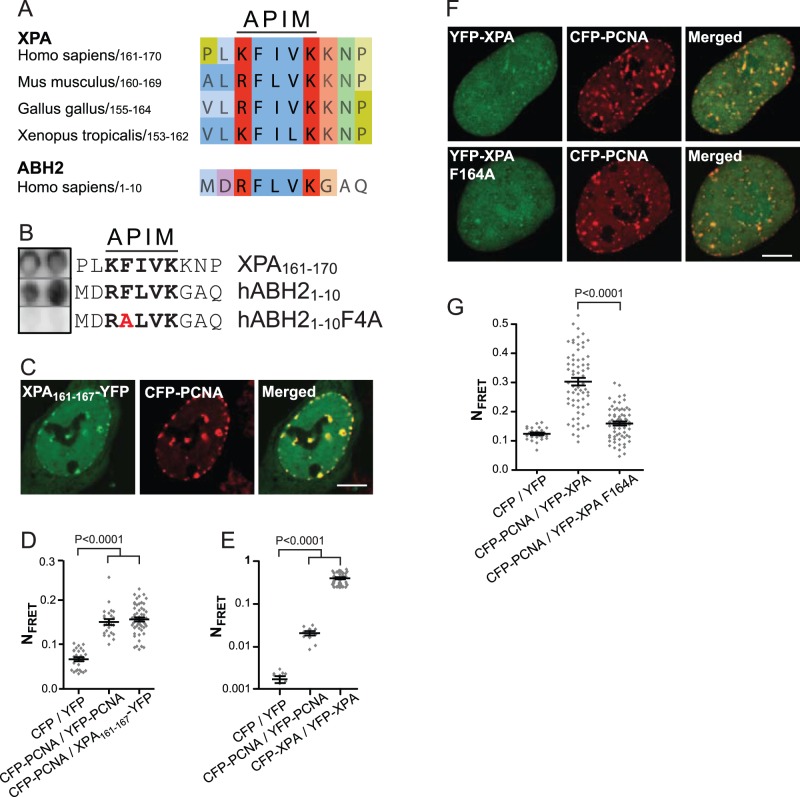
The APIM sequence in XPA is sufficient and necessary for interaction with PCNA. (A) Sequence alignment of the APIM sequence in XPA (aa 161–170 in human XPA) from different species compared with the APIM sequence in hABH2. The colors are given by Clustal X. (B) Dot blot with the human XPA APIM-peptide. The hABH2 APIM-peptide and its mutant are included as positive and negative controls, respectively (also used in [27]). Grey lines: dots from the same blot. (C) Images of YFP-tagged XPA_161−167_ co-expressed with CFP-tagged PCNA in live cycling HeLa cells. Yellow dots in the merged picture illustrate colocalization. Bar: 5 µM. (D and E) N_FRET_ measurements in HeLa cells. Detector gain: 800 (YFP), 700 (CFP), 700 (FRET) (D) and 700 (YFP), 800 (CFP), 700 (FRET) (E). CFP/YFP (vectors only) and CFP-PCNA/YFP-PCNA were used as negative and positive controls, respectively (mean ± SEM, n = 24–53 in D and n = 10–34 in E). (F) Overexpressed tagged proteins in live cycling XPA^−/−^ cells. Yellow dots in the merged picture illustrate colocalization. Bar: 5 µM. (G). N_FRET_ measurements in XPA^−/−^ cells. Detector gain: 800 (YFP), 700 (CFP), 700 (FRET) (mean ± SEM, n = 25–66). The P-values (D, E and G) are derived by unpaired t-test.

APIM in XPA is phylogenetically conserved (Figure 2A). Notably, in mouse XPA the APIM sequence is identical to the hABH2 APIM sequence. A dot blot with the XPA APIM-peptide was compared with the hABH2 APIM-peptide (positive control) and a peptide in which an A was substituted for the conserved F in the second APIM position (negative control) [27]. We found that binding of the XPA APIM-peptide to PCNA was equal to binding of the hABH2 APIM-peptide to PCNA (Figure 2B). Subsequent in vivo studies similarly showed that when a peptide containing the XPA APIM-sequence was fused to the YFP protein (XPA_161−167_-YFP), the fusion protein colocalized and generated positive FRET with CFP-PCNA (Figure 2C and D, respectively), indicating a direct interaction between APIM in XPA and PCNA.

We previously found that mutation in the completely conserved F residue in APIM reduced colocalization and/or FRET with PCNA [27]. However, there was no detectable difference in colocalization or FRET between XPA wt and XPA where F164 in APIM was mutated to A in HeLa cells (unpublished data). This could be caused by XPÁs reported ability to form dimers [24], enabling untagged endogenous XPA to bridge the overexpressed mutated XPA to PCNA. Dimerization of XPA was confirmed by FRET analysis (Figure 2E), thus next we co-expressed YFP-tagged wt or mutant XPA with CFP-PCNA in XPA^−/−^ cells to avoid this problem and performed FRET analysis. XPA F164A still colocalized with PCNA (Figure 2F); nevertheless, we found a significant reduction in FRET between XPA F164A and PCNA compared to between XPA wt and PCNA in these cells (Figure 2G). This strongly indicates that XPA binds directly to PCNA through its APIM sequence, and that the persistent colocalization in XPA^−/−^ cells is due to indirect binding via other proteins than XPA.

### Complete Reconstitution of XPA^−/−^ Cells Requires XPA with an Intact APIM Sequence

To study the functionality of the XPA-PCNA interaction, we next examined whether a reduced interaction between XPA and PCNA affected the UVR sensitivity. We applied low doses of UV-B that do not induce DSBs, but specifically induce 6-4 PPs and CPDs (see below and Figure S3).

**Figure 3:**
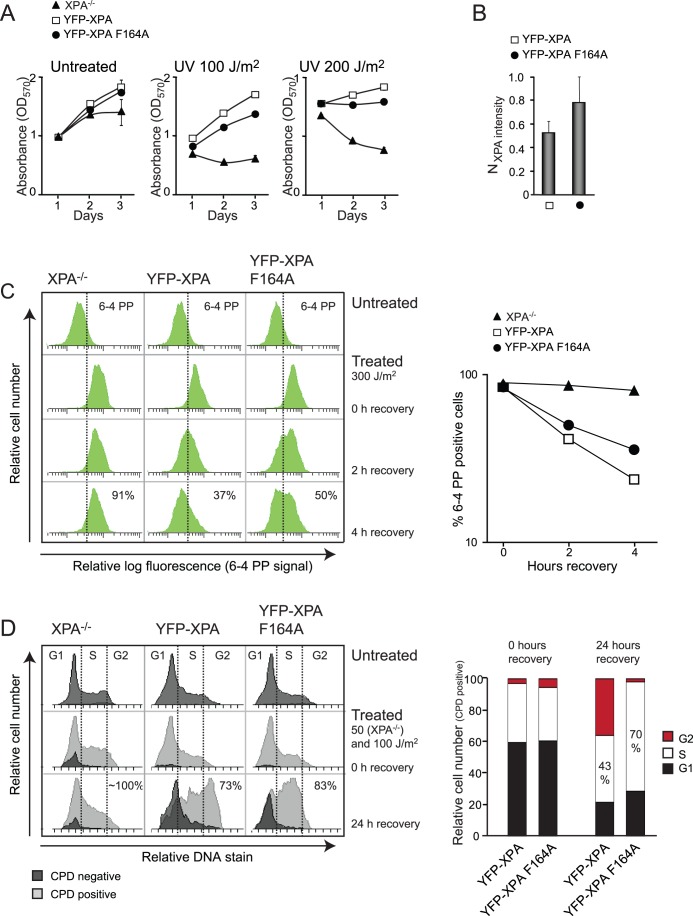
Complete reconstitution of XPA^−/−^ cells requires XPA with intact APIM. (A) Cell proliferation after UV-B treatment measured by MTT assay. The data is normalized against untreated day 1. One representative out of three experiments is presented. Data presented is the average of 6 wells ± SD. (B) Normalized XPA intensity measured by in-cell western (LI-COR Bioscience) (mean ± SD, n = 6). The XPA intensity is normalized against the DNA content using Draq5. (C) *Left panel:* Histograms of 6-4 PP positive cells, untreated, and 0, 2 and 4 h after UV-B. The cells with fluorescent intensity above the dashed line are defined as 6-4 PP positive. The numbers in the bottom row indicate % 6-4 PP positive cells 4 h after UVR. *Right panel:* Graphic presentation of data in left panel showing reduction of 6-4 PP positive cells as a function of time. (D) *Left panel:* Histograms illustrating cell cycle distribution of CPD positive and negative cells, untreated, 0 and 24 h after UV-B. Lower UVR-dose was applied for the XPA^−/−^ cells to avoid excessive apoptosis. The dashed lines separate the cell cycle phases. % CPD positive cells are given in bottom row. *Right panel*: Bars illustrating the relative cell-phase distribution of the CPD positive cells.

Cell survival assay (MTT) showed that XPA**^−/−^** cells reconstituted with XPA wt or XPA F164A fused to YFP displayed similar growth rates in absence of UVR. However, after UVR, cells expressing XPA F164A displayed a reduced growth rate compared to XPA wt, indicating a reduced tolerance to UVR when APIM is mutated (Figure 3A). Quantification by in-cell western demonstrated that the difference in UVR tolerance was not caused by lower expression levels of XPA F164A as the expression level of XPA F164A was slightly higher than for XPA wt (Figure 3B). These results indicate that although XPA colocalizes with PCNA in replication foci also in absence of DNA damage (Figure 1), the functionality of the interaction is only obvious after exposure to UVR.

We next exposed these cell lines to UVR and examined their repair capacities for 6-4 PP and CPD lesions and possible changes in cell phase distributions. Cells were harvested at various times after UVR exposure (allowing time for repair), stained with antibodies against 6-4 PPs and CPDs and analyzed by FACS. Our results show that cells reconstituted with XPA F164A repaired 6-4 PPs slower than cells reconstituted with XPA wt (50% versus 37% of the cells contain unrepaired 6-4 PPs 4 h after UVR, respectively), although not as slow as the XPA^−/−^ cells (91%) (Figure 3C). The graph in the right panel of Figure 3C compares the repair rates of 6-4 PPs for the different cell lines. The repair of 6-4 PPs was reduced in all phases of the cell cycle (Figure S2A), suggesting that the XPA-PCNA interaction is important for “overall” NER and not only for post-replicative NER in S phase. Likewise, removal of CPDs was also reduced in cells reconstituted with XPA F164A compared to cells reconstituted with XPA wt, i.e. contained more CPDs 24 h after UVR (83% versus 73%, respectively, Figure 3D, left panel). Moreover, more CPD positive cells expressing XPA F164A were arrested in S phase 24 h after UVR than cells expressing XPA wt (70% versus 43%, respectively, Figure 3D, right panel). In contrast, more CPD positive cells expressing XPA wt were arrested in G2. This difference in cell cycle arrest was most pronounced 24 h after UVR, but could also be detected after 48 h (Figure S2B). Difference in repair rates of CPDs between the cell lines cannot exactly be determined due to a combination of i) slow repair of CPDs, ii) proliferation, hence dilution of CPDs, and iii) because the proliferation rate at this UV-dose is lower for the cells expressing XPA F164A compared to cells expressing XPA wt (Figure 3A, mid panel). Nevertheless, the significant initial S phase accumulation of CPD positive cells expressing mutant XPA supports a reduced NER in cells lacking the direct XPA-PCNA interaction. In summary, these results show that the direct interaction between XPA and PCNA via the APIM motif is required for efficient repair of UVR-induced DNA lesions.

### Cells Lacking a Functional XPA-PCNA Interaction Accumulate Stalled Replication Forks after UVR Exposure

**Figure 4:**
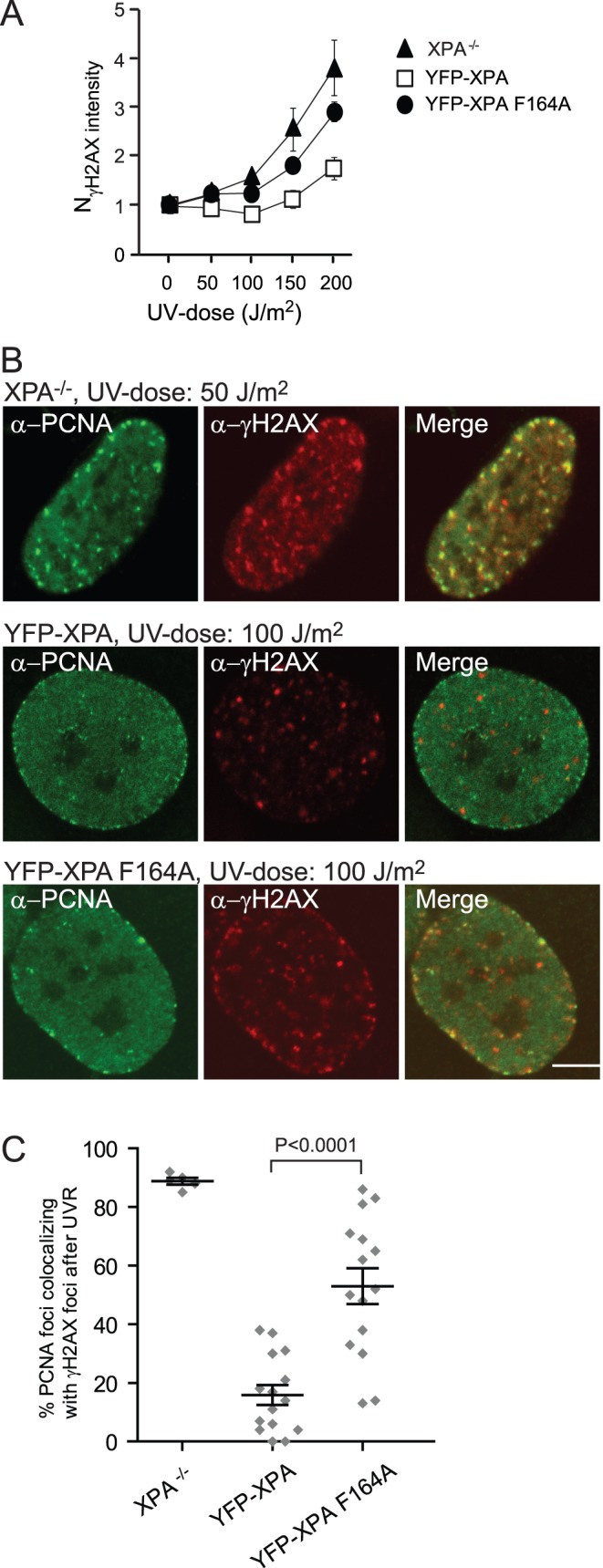
After UVR, cells complemented with APIM-mutated XPA accumulate γH2AX foci at the site of replication. (A) Normalized γH2AX intensity measured by in-cell western (LI-COR Bioscience) (mean ± SD, n = 4) 24 h after exposure to UV-B. The γH2AX intensity is normalized against the DNA content using Draq5 and the intensity of untreated cells. (B) Images of immunostained cells. The cells were exposed to UV-B 24 h prior to fixation. Lower UVR-dose was applied for the XPA^−/−^ cells to avoid excessive apoptosis. Bar: 5 µm. (C) Fractions of replication foci (PCNA) colocalizing with γH2AX. Each dot represents one cell, on average 35 foci were counted in each cell (mean ± SEM, n = 5 and 15). The P-value is derived by unpaired t-test. Only cells resembling S phase cells and expressing comparable levels of the YFP constructs were included.

To elucidate whether the impaired progression through S phase of the XPA F164A expressing cells was caused by stalled replication forks or DSBs, we next fixed the cells 24 h after UVR and stained for phosphorylated histone gamma H2AX (γH2AX), a marker for DSBs and stalled replication forks [37]. In-cell western (Figure 4A) shows that, 24 h after UVR, cells without a functional XPA-PCNA interaction (XPA^−/−^ and XPA F164A expressing cells) were stained for γH2AX at lower UV-doses than cells expressing XPA wt in agreement with reduced repair. Confocal analysis of cells stained for PCNA and γH2AX showed that XPA^−/−^ and XPA F164A expressing cells had higher levels of PCNA foci colocalizing with γH2AX than cells expressing XPA wt, ∼90% and ∼50% for XPA^−/−^ and XPA F164A expressing cells respectively, versus ∼20% for the XPA wt expressing cells (Figure 4B and C). Next, to elucidate whether these γH2AX foci were stalled replication forks or DSBs, we stained for RAD51 previously shown to localize at DSBs at collapsed replication forks [38]. Our cells treated with hydroxy urea overnight stained positively for RAD51 at replication foci (Figure S3A). However, we found that, unlike the γH2AX foci, the number of RAD51 foci did not increased upon radiation with these UV-doses and that the few RAD51 foci identified did not colocalize with replication foci (unpublished data and Figure S3B). Thus, the γH2AX foci colocalizing with PCNA most likely represent stalled replication forks, not DSBs.

Our results show that XPA interacts with PCNA close to the replication fork also in absence of UVR, however, the consequence of impaired direct interaction is only detected after damage. Recently, the ZRANB3 translocase was shown to interact with PCNA via both APIM and PIP at sites of replication in undamaged cells, but that the interaction was enhanced after DNA damage resulting in polyubiquitination of PCNA [32]. An APIM-YFP fusion protein pulled down PCNAs enriched in a subset of posttranslationally modified forms [27]. Therefore, it is tempting to speculate that the high affinity interaction between XPA and PCNA is found mainly between a posttranslationally modified PCNA and XPA. This remains to be elucidated, however, no obvious ubiquitination (mono or poly), or SUMOylation interaction domains could be identified in XPA by sequence analysis (data not shown).

**Figure 5:**
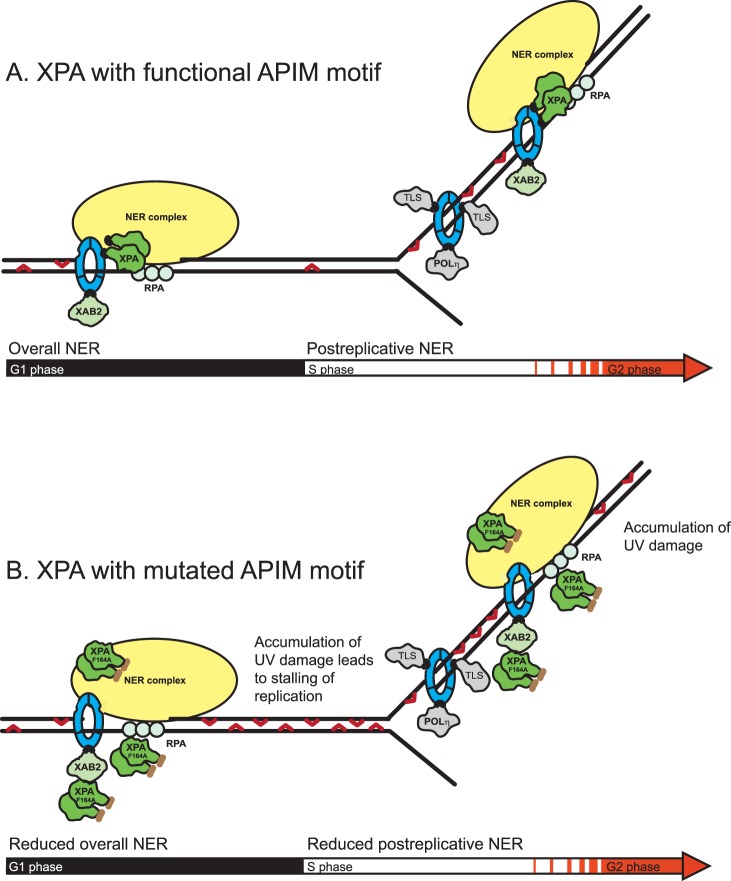
Model describing the role of direct XPA-PCNA interaction for efficient NER after UVR. To clarify the essence of our hypothesis, only the XPA dimer, XAB2, and RPA of the NER proteins are specified, and the NER complex (yellow) represents the other NER proteins in the model. The grey proteins mark proteins containing the PIP-box, the green mark proteins containing APIM, the blue donut marks PCNA and the red hooks mark 6-4 PPs and CPDs. (A) Optimal NER. (B) Reduced NER due to mutated APIM sequence in XPA.

A working model explaining our findings is shown in Figure 5. Because our experiments detect the functionality of the XPA-PCNA interaction only after UVR, the model illustrates the importance of direct XPA-PCNA interaction for efficient NER of UVR induced DNA damage. XPA and PCNA interact in complexes including the hitherto unidentified post-replicative NER complex (Figure 5A). This direct interaction is necessary for optimal NER throughout the cell cycle. Under normal conditions, e.g. after solar UVR, bypass of UV lesions is found to be important as illustrated by the severe phenotype of XP-Variant (XPV) patients [39]. However, after bypass the DNA lesions persist; hence repair systems for rapid removal of these frequent DNA lesions likely exist. Based on our data showing that XPA, XPF, XPD, XPC, and XPG are in the close proximity of newly replicated DNA and/or colocalize with PCNA in replication foci and that impaired XPA-PCNA interaction results in reduced NER also in S phase, we suggest that this is a mechanism for efficient postreplicative NER in S phase repairing lesions bypassed by TLS polymerases. If the DNA damage load is high, an initial pause in S phase will normally be followed by a late S/G2 arrest in order to repair all the bypassed lesions. This is observed for the XPA wt expressing cells 24 hours after UVR exposure (Figure 3D). Mutation in the APIM sequence in XPA impairs the direct interaction with PCNA; however, XPA still colocalizes with PCNA. This is likely due to indirect binding via RPA or other proteins in the NER complex for example XPA binding protein 2 (XAB2) which also contains APIM (Figure 5B). Nevertheless, an impaired direct APIM-mediated interaction between XPA and PCNA results in decreased NER efficiency. This leads to excessive levels of UVR induced DNA lesions that must be bypassed by TLS, resulting in an enhanced S phase and replication arrest as shown in Figure 3D and 4A-C. Reduced post-replicative NER efficiency likely also leads to higher levels of unrepaired DNA lesions in the subsequent replication. This is supported by our results showing that, even 48 h after UVR, the level of CPDs and cells stalled in S phase is enhanced in cells expressing the mutant XPA (Figure S2B). The reason for the lack of detectable G2 arrest in these cells is unknown and beyond the scope of this paper, but a connection between XPA and the checkpoint proteins ATR and Chk1 has previously been described [40].

### Conclusions

We have identified a functional direct interaction between XPA and PCNA mediated through the newly discovered APIM sequence. XPA with disrupted APIM sequence fails to fully rescue XPA^−/−^ cells after UVR; here we show reduced cell growth, reduced NER efficiency and an increased S phase arrest. We detect a direct interaction between XPA and PCNA and presence of XPA, XPF, and XPD in the replisome. These results suggest an important function of NER in post-replicative repair.

## Materials and Methods

### Expression Constructs

Cloning of the fluorescently tagged expression construct pCFP-PCNA is described [41]. The pYFP- and pCFP-XPA constructs were made by switching YFP and CFP tags with the His9-HA-GFP tag (NheI/BsrGI fragment) in pHis9-HA-GFP-XPA [42], a kind gift from Dr. Wim Vermeulen (Department of Cell Biology and Genetics, Rotterdam). pYFP-XPA was used as a template to design YFP-XPA F164A by site-directed mutagenesis according to the QuickChange II instruction manual (Stratagene). The Met-XPA_161−167_-YFP (called XPA_161−167_-YFP) construct was made by annealing oligos with NotI overhang, followed by ligation into pYFP-N1 mutated in the ATG codon. pYFP-XPG was generated by PCR amplification of XPG from pVL1392-XPG, a kind gift from Dr. Richard D. Wood, (Department of Molecular Carcinogenesis, Houston Texas) [43] and cloned into pYFP-C1 (Clonetech Laboratories, Inc) (XhoI/XmaI). pYFP-XPC was generated from pGFP-XPC (a kind gift by Dr. Wim Vermeulen) by switching tag (AgeI/HpaI and XmaI/HpaI for pYFP N1 and pGFP-XPC respectively) [44].

### Cell Lines

HeLa cells (ATCC CCL-2) transiently expressing fluorescently tagged proteins were prepared and cultured as described [27]. SV40 transformed XP-A fibroblast cells, (XPA deficient, XPA^−/−^) (Coriell Institute GM04429) were grown in alpha-MEM with the same supplements as for HeLa cells. XPA^−/−^ cells stably expressing YFP-XPA and YFP-XPA F164A were made by prolonged culturing in selective medium (G418) followed by cell sorting as described [27]. HEK293 cells (ATCC CRL-15B) were cultured as HeLa cells, except for 20% (instead of 10%) FBS to ensure that the cells were actively replicating at the time of iPOND.

### Immunofluoresence Staining

Cells were fixed with 2% paraformaldehyde and permeabilized with cold methanol. The cells were washed/blocked with 2% FCS in PBS prior to incubation with antibody against (α) PCNA (Abcam (ab)18197 or Santa Cruz biotechnology Inc., PC10), α-XPA (ab2352), α-γH2AX phospho S131 (ab2893), and α-RAD51 (Santa Cruz, H92) for 120 min at 37°C or overnight at 4°C. The cells were washed and incubated with Alexa fluor 532 goat α-mouse and Alexa fluor 647 goat α-rabbit (Invitrogen) for 45 min at 37°C, followed by confocal imaging.

### Confocal Imaging

Live HeLa and XPA^−/−^ cells were examined 16–24 h after transient transfection (by Fugene 6 or Fugene HD (Roche Inc.) according to the manufacturer’s recommendations) with the CFP/YFP fusion constructs or after immunofluorescence staining. The fluorescent images were acquired using a Zeiss LSM 510 Meta laser scanning microscope equipped with a Plan-Apochromate 63×/1.4 oil immersion objective. The images were acquired in the growth medium of the cell, with the stage heated to 37°C, using the Zeiss LSM 510 software. CFP was excited at λ = 458 nm and detected at λ = 470–500 nm and YFP was excited at λ = 514 nm and detected at λ = 530–600 nm as in [27]. The immunostained cells were excited at λ = 543 nm and 633 nm laser lines and detected at λ = 560–615 nm and λ>650 nm for Alexa fluor 532 and 647, respectively. When YFP-XPA was imaged together with immunostained proteins, YFP was excited at λ = 488 nm and detected at λ = 505–570 nm to limit bleed through. The thickness of the slice was 1 µm. All images were acquired with consecutive scans to avoid bleed though. No image processing, except contrast and intensity adjustments, were performed.

### Fluorescence Resonance Energy Transfer (FRET) Analysis

FRET occurs if tags with spectral overlap (here: YFP and CFP) are less than 100 Å (10 nm) apart [36]. We detected FRET using the sensitized emission method, measuring acceptor (YFP) emission upon donor (CFP) excitation as in [27]. FRET was scored when the intensity of emitted light from YFP after excitation of the CFP fluorochrome was stronger than the light emitted by CFP or YFP-tagged proteins alone, after excitation with CFP lasers (false FRET), given by the equation: FRET =  I2– I1 (ID2/ID1) - I3 (IA2/IA3). FRET >0 was normalized for expression levels using the equation: N_FRET_  =  FRET/(I1 × I3)1/2 [45], [46]. N_FRET_ was calculated from mean intensities (I) within a region of interest (ROI) containing more than 25 pixels where all pixels had intensities below 250. Channel 1 (CFP) and 3 (YFP) were measured as described for confocal imaging, and channel 2 (FRET) was excited with λ = 458 nm and detected at λ = 530–600 nm. ID1, D2, and IA2, A3 were determined for cells transfected with CFP and YFP constructs only, with same settings and same fluorescence intensities as co-transfected cells (I1 and I3).

### FACS Analysis

For measurements of 6-4 PPs and CPDs (modified from [47]), cells were fixed in ice-cold 100% methanol and treated with 0.5% Triton-X/2M HCl for 10 min at room temperature followed by washing with 0.1 M Na_2_B_4_O_7_ (pH 9.0) and PBS. The cell pellets were then incubated in 300 µl RNAse (100 µg/ml in PBS) at 37°C for 60 min before incubation with α- 6-4 PP and -CPD (64M-2 and TDM2, Cosmo Bio) at 4°C overnight. The antibodies were diluted in PBS-TB (1% BSA/0.25% Tween-20/PBS). The cells were then washed in PBS-TB, followed by incubation with Alexa fluor 405 goat α-mouse (Invitrogen) for 60 min at room temperature. Finally, the cells were washed in PBS-TB and resuspended in PBS with Propidium Iodide (PI, Molecular Probes) at a final volume of 5 µg/ml. PI was excited at λ = 488 nm and detected at λ = 575 nm. Alexa fluor 405 goat α-mouse was excited at λ = 407 nm and detected at λ = 450 nm. Cell cycle fractions, 6-4 PPs and CPDs were determined by using BD FACSAria and the BD FACSDiva software (BD Biosciences). Data presented is one representative out of 5 (UV lesions) and 8 (cell cycle) individual experiments revealing the same trend.

### Preparation of Cell Extract and Co-immunoprecipitation (co-IP)

Fractionated cell extracts from HeLa cells were prepared as described [27]. Importantly, the sonicated pellets (containing nuclei) were excessively treated with a DNAse/RNAse cocktail (2 µl Omnicleave Endonuclease (200 U/µl Epicentre Technologies, WI), 1 µl DNAse (10 U/µl, Roche Inc.), 1 µl Benzonase (250 U/µl, Novagene, Ge), 1 µl Micrococcal Nuclease (100–300 U/mg, Sigma-Aldrich) and 10 µl RNAse (2 mg/ml, Sigma-Aldrich) per 30 mg cell extract, at 37°C for 1 hour. Monoclonal α-PCNA (PC10) and an in-house purified polyclonal α-GFP were covalently linked to protein-A paramagnetic beads (Dynal) according to a procedure from New England Biolabs Inc (from now on called α-PCNA and α-YFP beads). 1500 µg of each fraction was incubated with 20 µl α-PCNA or 10 µl α-YFP beads during constant rotation at 4°C overnight (IP). For the IP with endogenous proteins, α-YFP beads were used as a negative control to rule out unspecific binding to the beads.

### Isolation of Proteins on Nascent DNA (iPOND)

The iPOND was performed essentially as described in [34]. Shortly, HEK293 cells (2×10^8^ cells per sample) were pulsed with media containing 5-ethynyl-2′-deoxyuridine (EdU) (10 µM, Invitrogen) for 5 to 15 min (pulse). For the pulse-chase experiment, the EdU was replaced with media containing thymidine for 30 min (10 µM, Sigma-Aldrich). Media containing DMSO was used as a negative control (incubated for 15 min, termed 0 min EdU in the Figure). After pulse and pulse-chase, cells were cross-linked in formaldehyde/PBS (1%) for 20 min at room temperature, quenched using 0.125 M glycine, and washed three times in cold PBS. The cell pellets were frozen at −80°C, and then resuspended in 0.25% Triton-X/PBS. Pellets were washed once with 0.5% BSA/PBS and once with PBS. Cells were incubated in click reaction buffer for 1–2 h at a concentration of 1×10^8^ cells per 5 ml of click reaction buffer (2 mM CuSO_4_, 10 µM biotin-azide, 10 mM Sodium ascorbate). Cell pellets were then washed once with 0.5% BSA/PBS and once with PBS, resuspended in lysis buffer (1% SDS, 50 mM Tris (pH 8.0), 1 µg/ml leupeptin, and 1 µg/ml aprotinin) and sonicated. Samples were centrifuged, filtered through an 80-µm nylon mesh, and diluted 1∶1 with PBS containing 1 µg/ml leupeptin and 1 µg/ml aprotinin prior to purification. Streptavidin–agarose resin (100 µl resin per 2×10^8^ cells, Novagen) was washed twice in lysis buffer and once in PBS. Washed resin was incubated with the samples for 16–20 h at 4°C. The resin was washed once with lysis buffer, once with 1 M NaCl, and then twice with lysis buffer. Captured proteins were eluted and cross-links were reversed in (1∶1) SDS Laemmli sample buffer (0.4 g SDS, 2 ml 100% Glycerol, 1.25 ml 1M Tris pH 6.8, 0.01 g Bromphenol blue, and 0.2 M DTT in 8 ml H_2_O) by incubating for 25 min at 95°C. Proteins were resolved on SDS-PAGE and detected by western blot.

### Western Blot (WB), in-cell Western and Dot-blot Analysis

For WB α-PCNA (PC10), α-XPA (ab65963), α-XPF (ab17798), α-XPD (ab54676), and α-Histone H3 (ab1791) were used and the procedure conducted as described [27]. Expression of YFP-XPA constructs were measured parallel to the cell survival assay and accumulation of γH2AX was verified 24 h after UVR, using In-Cell Western assay by the Odyssey Infrared Imaging System according to the provider’s protocol (LI-COR Bioscience), and using α-XPA (ab2352), α-γH2AX (ab2893), DRAQ5 (DNA stain, Biostatus), and IRDye 800CW Goat Anti-Mouse Secondary Antibody (LI-COR Bioscience). The cell plates were scanned in the 700 nm and 800 nm laser channels using the Odyssey Imager. The fluorescent intensity in the 800 nm channel (XPA or γH2AX signal) was normalized against the 700 nm channel (DNA stain ≈ cell number) after background subtraction for each well. The data presented is an average from 5 wells. A dot blot containing 28 nmol peptides was performed as described [27] using 1 µg/ml recombinant PCNA and developed with α-PCNA (PC10) as for WB.

### Cell Survival Assay

XPA^−/−^ cells, untransfected and stably expressing YFP-XPA and YFP-XPA F164A, were seeded into 96 well plates (4000 cells/well) and incubated for 4 h. UV-B exposure: medium was replaced with 50 µl PBS and the plates were exposed to various doses of UV-B (Vilber Lourmat, Bio Spectra V5, 312 nm). Cells were harvested every day for the next four days using the MTT (3-(4.5-Dimethylthiazol-2-yl)-2.5 diphenyl-tetrazolium bromide) assay. OD was measured at 570 nm, and the average from at least 4 wells was used to calculate cell survival.

## Acknowledgments

We wish to thank Siri Bachke and Caroline Danielsen Søgaard for technical assistance, Dr. Morten Rye for search for binding domains in XPA, Dr. David Cortez and Bianca Sirbu (Department of Biochemistry, Nashville Tennessee) for sharing unpublished iPOND details, Dr. Orlando Schärer (Department of Pharmacological Sciences, NY) for constructive contributions to the manuscript preparations.

## Supporting Information

Figure S1 XPC and XPG colocalize with PCNA in replication foci. Confocal fluorescent images of YFP-tagged XPC and XPG co-expressed with CFP-PCNA in live, freely cycling, untreated XPA−/− cells.
(EPS)

Figure S2 Repair of 6–4 PP and CPD. (A) Graph showing the level of 6–4 PP positive cells as a function of recovery time in all phases of the cell cycle (G1, S and G2) in XPA−/− (▴) and XPA−/− stably expressing YFP-XPA (□) and YFP-XPA F164A (•). The cells are treated with UVR (300 J/m2 UV-B), and the data is extracted from the experiment shown in Figure 3C. The 6–4 PP levels are normalized against the 6–4 PP levels at time 0 h after UVR. (B) Histograms representing the cell cycle distribution of CPD negative and positive cells. Cells were analyzed 0, 24, 48, and 72 h after UVR (100 J/m2 UV-B). Untreated cells are included as a negative control. CPD negative and positive cells are shown in dark and light grey, respectively. Histograms for unexposed cells, and UV-exposed cells at 0 and 24 h are the same as shown in Fig. 3D.
(EPS)

Figure S3 RAD51 foci in hydroxy urea (HU)- and UV-exposed cells. (A) Images of XPA−/− cells stably expressing YFP-XPA stained for PCNA and RAD51 after HU exposure (10 mM). Colocalization between PCNA and RAD51 in these cells is positive control for (B), showing that RAD51 functions as a marker for collapsed replication forks, i.e. DSBs. (B) Images of XPA−/− cells stained for PCNA and RAD51 24 h after UVR (50 J/m2 UV-B). Lack of colocalization between PCNA and RAD51 indicate that these cells do not contain DSBs at the site of replication, i.e. collapsed replication forks.
(EPS)
